# (4-Butyl-1-ethyl-1,2,4-triazol-5-yl­idene)[(1,2,5,6-η)-cyclo­octa-1,5-diene](tri­phenyl­phosphane)rhodium(I) tetra­fluorido­borate

**DOI:** 10.1107/S2414314624000609

**Published:** 2024-01-26

**Authors:** Timothy G. Lerch, Michael Gau, Daniel R. Albert, Edward Rajaseelan

**Affiliations:** a Department of Chemistry, Millersville University, Millersville, PA 17551, USA; b Department of Chemistry, University of Pennsylvania, Philadelphia, PA 19104, USA; University of Aberdeen, United Kingdom

**Keywords:** crystal structure, rhodium, N-heterocyclic carbenes, cationic complexes

## Abstract

In the title triazole-based N-heterocyclic carbene rhodium(I) cationic complex with a tetra­fluorido­borate counter-anion, [Rh(C_8_H_12_)(C_8_H_15_N_3_)(C_18_H_15_P)]BF_4_, which crystallizes with two cations and two anions in the asymmetric unit, the Rh center has a distorted square-planar coordination geometry with expected bond distances. Several nonclassical C—H⋯F hydrogen-bonding inter­actions help to consolidate the packing. Two of the F atoms of one of the anions are disordered over adjacent sites in a 0.814 (4):0.186 (4) ratio.

## Abstract 2

In the title triazole-based N-heterocyclic carbene rhodium(I) cationic complex with a tetra­fluorido­borate counter-anion, the Rh center has a distorted square-planar coordination geometry with expected bond distances.

## Graphical abstract

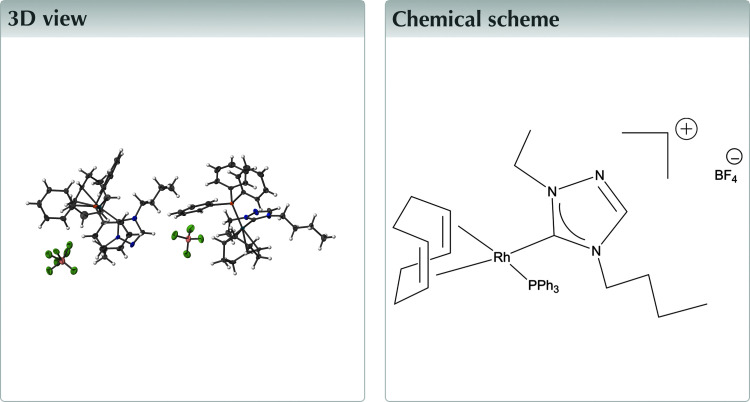

## Structure description

N-Heterocyclic carbenes (NHCs) have become important alternatives to phosphines as ancillary ligands in transition-metal chemistry, synthesis, and in homogeneous catalysis (Cazin, 2013 ▸; de Frémont *et al.*, 2009 ▸; Díez-González *et al.*, 2009 ▸; Rovis & Nolan, 2013 ▸; Ruff *et al.*, 2016 ▸; Zuo *et al.*, 2014 ▸). Their catalytic activity in the transfer hydrogenation of ketones and imines has also been studied and reported (Albrecht *et al.*, 2002 ▸; Gnanamgari *et al.*, 2007 ▸). The use of N-heterocyclic carbenes as ligands and having different substituents on the N atoms, enables the variation of the steric and electronic properties of the metal center (Díez-González *et al.*, 2007 ▸; Gusev, 2009 ▸). Many imidazole- and triazole-based NHC rhodium and iridium complexes have been synthesized and structurally characterized (Herrmann *et al.*, 2006 ▸; Wang & Lin 1998 ▸; Chianese *et al.*, 2004 ▸). We continue to synthesize new imidazole- and triazole-based NHC complexes of rhodium and iridium to study the effect of different substituents on the NHC and the other ligands coordinated to the metal in transfer hydrogenation reactions (Nichol *et al.*, 2009 ▸, 2010 ▸, 2011 ▸, 2012 ▸; Idrees *et al.*, 2017*a* ▸,*b* ▸; Rood *et al.*, 2021 ▸; Rushlow *et al.*, 2021 ▸; Newman *et al.*, 2021 ▸; Castaldi *et al.*, 2021 ▸; Maynard *et al.*, 2023 ▸).

**Figure 1:**
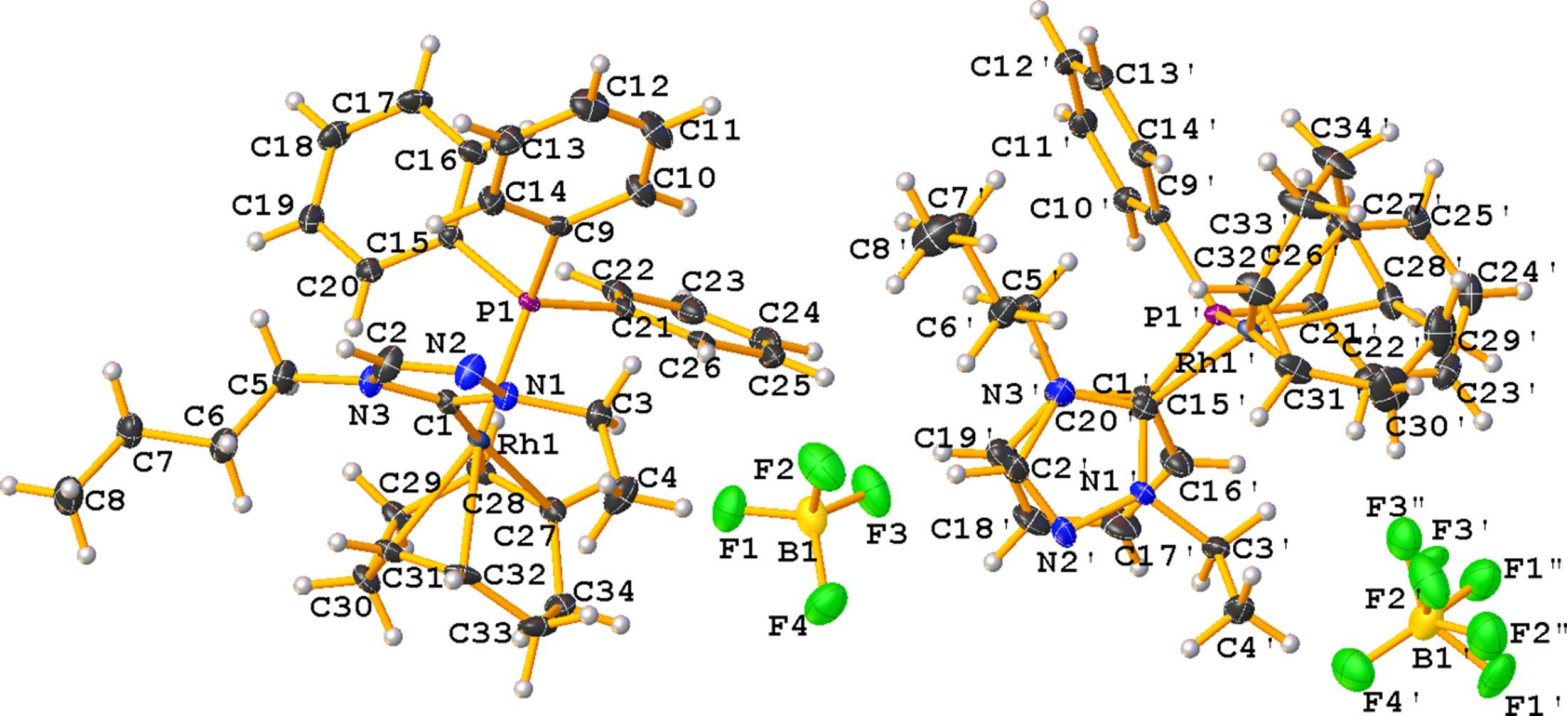
The mol­ecular entities of the title compound (**4**), with displacement ellipsoids drawn at the 50% probability level.

**Figure 2:**
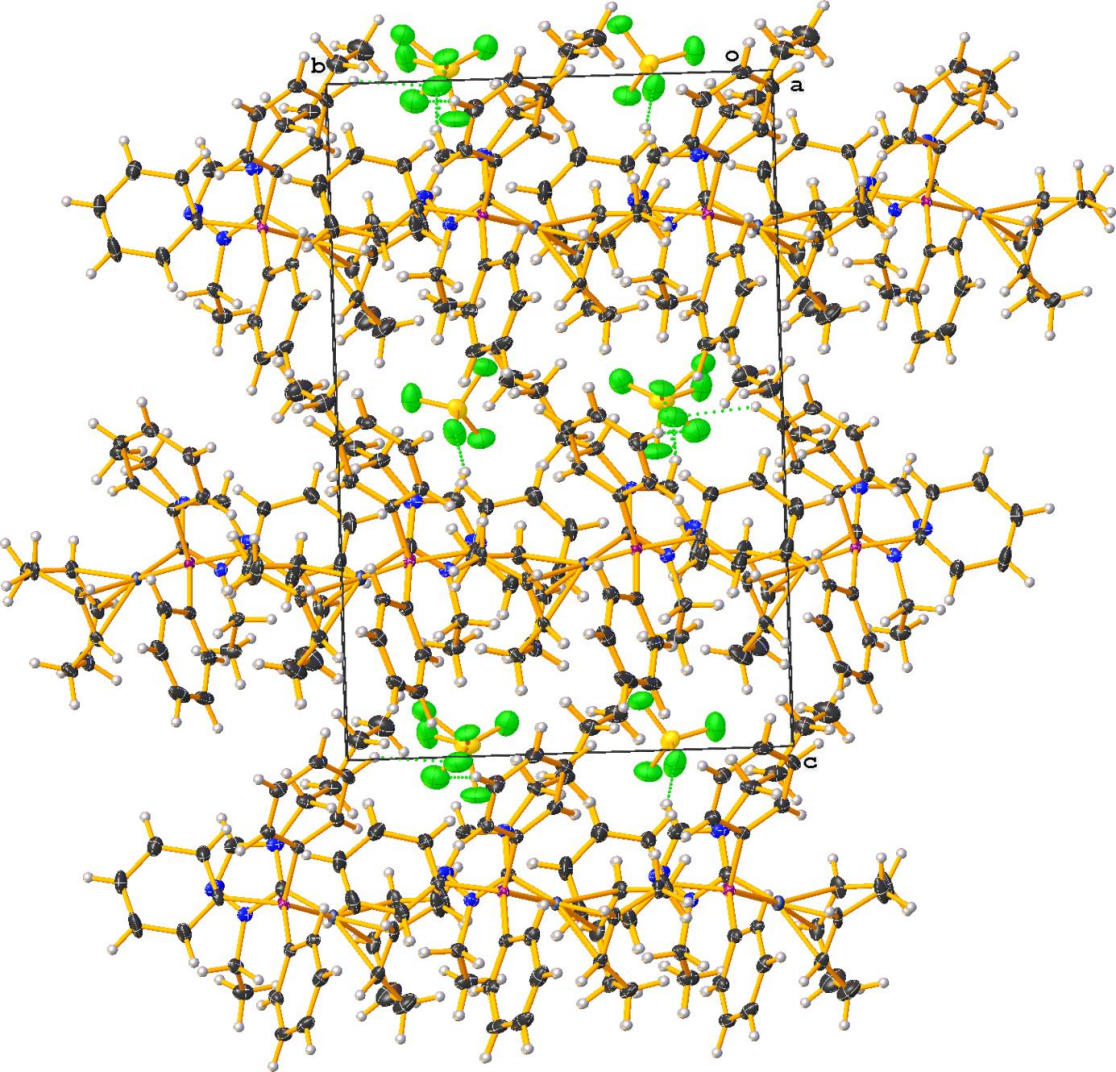
Crystal packing diagram of the title compound (**4**), viewed along the *a*-axis direction. C—H⋯F hydrogen bonds are shown as dotted green lines.

The mol­ecular structure of the title complex, [Rh(C_8_H_12_)(C_8_H_15_N_3_)(C_18_H_15_P)]BF_4_ (**4**), comprises an Rh^I^ cation complex and a tetra­fluorido­borate counter-anion, illustrated in Fig. 1 ▸. No solvent mol­ecules were found in the structure. The compound crystallizes in the monoclinic space group *Pc* with two cations (A containing Rh1 and B containing Rh1′) and two anions in the asymmetric unit. The coordination sphere around the Rh^I^ ion, formed by the bidentate (1,2,5,6-η)-cyclo­octa-1,5-diene ligand (C_8_H_12_; COD), the C_8_H_15_N_3_ NHC ligand and the tri­phenyl­phosphane ligand, results in a distorted RhCP(η^2^C=C)_2_ square-planar geometry. The carbene atom, C1/C1′, deviates from the expected *sp* ^2^ hybridization in that the N1—C1—N3 (cation A) and N1′—C1′—N3′ (cation B) bond angles are 103.3 (3) and 102.9 (3)°, respectively. Other selected bond lengths and angles in cations A and B are Rh—C_NHC_ = 2.034 (4) and 2.038 (4) Å, Rh—P = 2.3162 (9) and 2.3135 (9) Å, and C_NHC_—Rh—P = 91.84 (10) and 90.63 (10)°, respectively. Fig. 2 ▸ shows the packing diagram of the title compound viewed along [100], with nonclassical C—H⋯F hydrogen bonds shown as dotted green lines; the CH moieties for these inter­actions arise from both the N-heterocyclic carbene and phenyl moieties (Table 1 ▸). The extremely short H⋯F contact distance of 1.81 Å for C2—H2⋯F1 is probably an artefact of disorder of the F atom.

## Synthesis and crystallization

**Figure 3:**
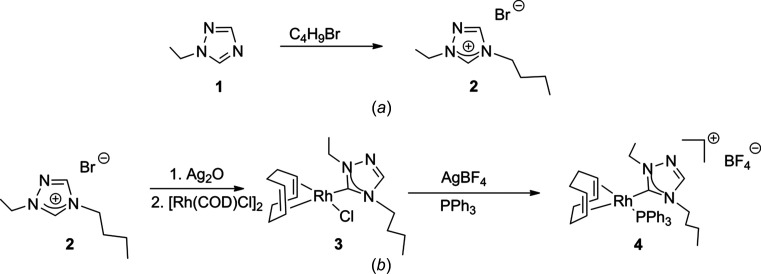
Reaction schemes for synthesis of (*a*) the triazolium salt (**2**) and (*b*) title compound (**4**).

1-Ethyl-1,2,4-triazole (**1**) was purchased from Matrix Scientific. All other compounds used in the syntheses were obtained from Sigma–Aldrich and Strem and used as received; all syntheses were performed under a nitro­gen atmosphere. The reaction scheme for the synthesis of the title compound is shown in Fig. 3 ▸. NMR spectra were recorded at room temperature in CDCl_3_ on a 400 MHz (operating at 100 MHz for ^13^C and 162 MHz for ^31^P) Varian spectrometer and referenced to the residual solvent peak (δ in ppm). The title compound (**4**) was crystallized by slow diffusion of pentane into a CH_2_Cl_2_ solution.

**4-Butyl-1-ethyl-1,2,4-triazolium bromide (2):** 1-ethyl-1,2,4-triazole (**1**) (1.001 g, 10.30 mmol) and excess 1-bromo­butane (5.113 g, 37.31 mmol) were added to toluene (15 ml), and the mixture was refluxed in the dark for 48 h. After the mixture was cooled, the white solid was filtered off, washed with ether, and dried under vacuum (yield: 2.120 g, 88%). ^1^H NMR: δ 11.66 (*s*, 1H, N—C5H—N), 8.94 (*s*, 1H, N—C3H—N), 4.59 (*q*, 2H, N—CH_2_ of eth­yl), 4.55 (*t*, 2H, N—CH_2_ of but­yl), 1.98 (*m*, 2H, CH_2_ of but­yl), 1.64 (*t*, 3H, CH_3_ of eth­yl), 1.42 (*m*, 2H, CH_2_ of but­yl), 0.97 (*t*, 3H, CH_3_ of Bu). ^13^C NMR: δ 143.62 (N—C5—N), 142.71 (N—C3—N), 48.56 (N—CH_2_ of eth­yl), 48.34 (N—CH_2_ of but­yl), 31.98 (CH_2_ of but­yl), 19.48 (CH_2_ of but­yl), 14.21 (CH_3_ of eth­yl), 13.41 (CH_3_ of but­yl).

**(4-Butyl-1-ethyl-1,2,4-triazol-5-yl­idene)[(1,2,5,6-η)-cyclo­octa-1,5-diene]chlorido­rhodium (3):** triazolium bromide (**2**) (0.095 g, 0.406 mmol) and Ag_2_O (0.047 g, 0.203 mmol) were stirred at room temperature in the dark for 1 h in CH_2_Cl_2_ (10 ml). The mixture was then filtered through Celite into [Rh(cod)Cl]_2_ (0.100 g, 0.203 mmol), and stirred again in the dark for 1.5 h. The resulting solution was filtered through Celite and the solvent was removed under reduced pressure in a rotary evaporator. The yellow solid product (**3**) was dried under vacuum (yield: 0.158 g, 98%). ^1^H NMR: δ 7.86 (*s*, 1H, N—C3H—N), 4.75 (*q*, 2H, N—CH_2_ of eth­yl), 4.62 (*t*, 2H, N—CH_2_ of but­yl), 4.50 (*m*, 2H, CH of COD), 4.48 (*m*, 2H, CH of COD), 3.36–3.24 (*m*, 4H, CH_2_ of COD), 2.38–2.08 (*m*, 4H, CH_2_ of COD), 1.89 (*m*, 2H, CH_2_ of but­yl), 1.55 (*m*, 2H, CH_2_ of but­yl), 1.43 (*m*, 2H, CH_2_ of but­yl), 1.50 (*t*, 3H, CH_3_ of eth­yl), 1.05 (*t*, 3H, CH_3_ of but­yl). ^13^C NMR: δ 184.73 (*d*, Rh—C, *J*_C–Rh_ = 50.9 Hz), 141.76 (N—C3H—N), 99.50, 99.43, 99.32, 99.25 (CH of COD), 48.45 (N—CH_2_ of eth­yl), 47.91 (N—CH_2_ of but­yl), 33.10, 32.69, 32.61, 29.09 (CH_2_ of COD), 28.61 (CH_2_ of but­yl), 20.02 (CH_2_ of but­yl), 15.39 (CH_3_ of eth­yl), 13.69 (CH_3_ of but­yl).

**(4-Butyl-1-ethyl-1,2,4-triazol-5-yl­idene)[(1,2,5,6-η)-cyclo­octa-1,5-diene](tri­phenyl­phosphane)rhodium(I) tetra­fluorido­borate (4):** tri­phenyl­phosphane (0.104 g, 0.395 mmol) and AgBF_4_ (0.077 g, 0.395 mmol) were added to (**3**) (0.158 g, 0.395 mmol) in CH_2_Cl_2_ (15 ml). The solution was stirred in the dark for 1.5 h. The resulting mixture was filtered through Celite and the solvent was removed under reduced pressure. The bright-orange solid product (**4**) was dried under vacuum. Orange blocks suitable for data collection were recrystallized from **xxx solution [???]** (yield: 0.282 g, 99%). ^1^H NMR: δ 8.14 (*s*, 1H, N—C3H—N), 7.58–7.26 (*m*, 15H, H_arom_), 4.78 (*q*, 2H, N—CH_2_ of eth­yl), 4.51 (*t*, 2H, N—CH_2_ of but­yl), 4.43 (*m*, 2H, CH of COD), 4.36 (*m*, 2H, CH of COD), 3.92 (*m*, 2H, CH_2_ of COD), 3.77 (*m*, 2H, CH_2_ of COD), 2.56 (*m*, 2H, CH_2_ of COD), 2.46 (*m*, 2H, CH_2_ of COD), 2.26 (*m*, CH_2_ of but­yl), 1.92 (*m*, 2H, CH_2_ of but­yl), 1.22 (*t*, 3H, CH_3_ of eth­yl), 0.95 (*t*, 3H, CH_3_ of but­yl). ^13^C NMR: δ 180.96 (*d*, Rh—C, *J*_C–Rh_ = 49.3 Hz), 143.61 (N—C3H—N), 133.44–128.66 (C_arom_), 97.99, 97.82, 96.89, 95.19 (CH of COD), 48.63 (N—CH_2_ of eth­yl), 47.94 (N—CH_2_ of but­yl), 31.70, 31.59, 30.57, 30.55 (CH_2_ of COD), 30.19 (CH_2_ of but­yl), 20.04 (CH_2_ of but­yl), 13.97 (CH_3_ of eth­yl), 13.30 (CH_3_ of but­yl).^31^P NMR: δ 25.91 (*d*, Rh—P, *J*_P–Rh_ = 154 Hz).

## Refinement

All H atoms were placed geometrically and refined as riding atoms, with *U*_iso_(H) = 1.2*U*_eq_(C) or 1.5*U*_eq_(methyl C). Two of the F atoms are disordered over adjacent sites in a 0.814 (4):0.186 (4) ratio. Refinement details are summarized in Table 2 ▸.

## Supplementary Material

Crystal structure: contains datablock(s) I. DOI: 10.1107/S2414314624000609/hb4460sup1.cif

Structure factors: contains datablock(s) I. DOI: 10.1107/S2414314624000609/hb4460Isup2.hkl

CCDC reference: 2327318

Additional supporting information:  crystallographic information; 3D view; checkCIF report

## Figures and Tables

**Table 1:** Hydrogen-bond geometry (Å, °)

*D*—H⋯*A*	*D*—H	H⋯*A*	*D*⋯*A*	*D*—H⋯*A*
C2—H2⋯F3′^i^	0.95	2.37	3.263 (5)	157
C2—H2⋯F1"^i^	0.95	1.81	2.722 (13)	160
C17—H17⋯F2"^ii^	0.95	2.30	3.229 (17)	165
C2′—H2′⋯F3	0.95	2.19	3.002 (5)	142
C13′—H13′⋯F1"^iii^	0.95	2.36	3.163 (17)	142

Symmetry codes: (i) 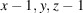 ; (ii) 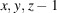 ; (iii) 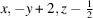 .

**Table 2:** Experimental details

Crystal data	
Chemical formula	[Rh(C_8_H_12_)(C_8_H_15_N_3_)(C_18_H_15_P)]BF_4_
*M*_r_	713.39
Crystal system, space group	Monoclinic, *P* *c*
Temperature (K)	100
*a*, *b*, *c* (Å)	14.1427 (4), 12.3145 (4), 18.8729 (5)
β (°)	98.442 (3)
*V* (Å^3^)	3251.30 (17)
*Z*	4
Radiation type	Mo *K*α
μ (mm^−1^)	0.63
Crystal size (mm)	0.24 × 0.11 × 0.09

Data collection	
Diffractometer	Rigaku XtaLAB Synergy-S
Absorption correction	Multi-scan (*CrysAlis PRO*; Rigaku OD, 2023 ▸)
*T*_min_, *T*_max_	0.873, 1.000
No. of measured, independent and observed [*I* > 2σ(*I*)] reflections	51545, 14495, 12922
*R*_int_	0.043
(sin θ/λ)_max_ (Å^−1^)	0.667

Refinement	
*R*[*F* ^2^ > 2σ(*F* ^2^)], *wR*(*F* ^2^), *S*	0.032, 0.079, 1.07
No. of reflections	14495
No. of parameters	825
No. of restraints	83
H-atom treatment	H-atom parameters constrained
Δρ_max_, Δρ_min_ (e Å^−3^)	0.89, −0.59
Absolute structure	Flack *x* determined using 5071 quotients [(*I* ^+^) − (*I* ^−^)]/[(*I* ^+^) + (*I* ^−^)] (Parsons *et al.*, 2013 ▸)
Absolute structure parameter	−0.015 (11)

Computer programs: *CrysAlis PRO* (Rigaku OD, 2023 ▸), *olex2.solve* (Bourhis *et al.*, 2015 ▸), *SHELXL2018* (Sheldrick, 2015 ▸), *OLEX2* (Dolomanov *et al.*, 2009 ▸) and *publCIF* (Westrip, 2010 ▸).

## full crystallographic data

### Crystal data

**Table:**

[Rh(C_8_H_12_)(C_8_H_15_N_3_)(C_18_H_15_P)]BF_4_	*F*(000) = 1472
*M**_r_* = 713.39	*D*_x_ = 1.457 Mg m^−^^3^
Monoclinic, *P**c*	Mo *K*α radiation, λ = 0.71073 Å
*a* = 14.1427 (4) Å	Cell parameters from 29059 reflections
*b* = 12.3145 (4) Å	θ = 2.2–28.3°
*c* = 18.8729 (5) Å	µ = 0.63 mm^−^^1^
β = 98.442 (3)°	*T* = 100 K
*V* = 3251.30 (17) Å^3^	Block, orange
*Z* = 4	0.24 × 0.11 × 0.09 mm

### Data collection

**Table:**

Rigaku XtaLAB Synergy-S diffractometer	12922 reflections with *I* > 2σ(*I*)
Detector resolution: 10.0 pixels mm^-1^	*R*_int_ = 0.043
ω scans	θ_max_ = 28.3°, θ_min_ = 2.2°
Absorption correction: multi-scan SCALE3 ABSPACK	*h* = −18→18
*T*_min_ = 0.873, *T*_max_ = 1.000	*k* = −14→16
51545 measured reflections	*l* = −25→23
14495 independent reflections	

### Refinement

**Table:**

Refinement on *F*^2^	Hydrogen site location: inferred from neighbouring sites
Least-squares matrix: full	H-atom parameters constrained
*R*[*F*^2^ > 2σ(*F*^2^)] = 0.032	*w* = 1/[σ^2^(*F*_o_^2^) + (0.0385*P*)^2^ + 0.0978*P*] where *P* = (*F*_o_^2^ + 2*F*_c_^2^)/3
*wR*(*F*^2^) = 0.079	(Δ/σ)_max_ = 0.001
*S* = 1.07	Δρ_max_ = 0.89 e Å^−^^3^
14495 reflections	Δρ_min_ = −0.59 e Å^−^^3^
825 parameters	Absolute structure: Flack *x* determined using 5071 quotients [(I+)-(I-)]/[(I+)+(I-)] (Parsons *et al.*, 2013)
83 restraints	Absolute structure parameter: −0.015 (11)
Primary atom site location: iterative	

### Special details

**Table:**

Geometry. All esds (except the esd in the dihedral angle between two l.s. planes) are estimated using the full covariance matrix. The cell esds are taken into account individually in the estimation of esds in distances, angles and torsion angles; correlations between esds in cell parameters are only used when they are defined by crystal symmetry. An approximate (isotropic) treatment of cell esds is used for estimating esds involving l.s. planes.

### Fractional atomic coordinates and isotropic or equivalent isotropic displacement parameters (Å^2^)

**Table:**

	*x*	*y*	*z*	*U*_iso_*/*U*_eq_	Occ. (<1)
Rh1	0.23043 (2)	0.54241 (2)	0.22149 (2)	0.01140 (11)	
P1	0.35044 (7)	0.66162 (7)	0.20155 (5)	0.0126 (2)	
N1	0.0963 (2)	0.7316 (2)	0.21225 (16)	0.0169 (6)	
N2	0.0233 (3)	0.7901 (3)	0.17234 (17)	0.0241 (7)	
N3	0.0703 (2)	0.6506 (2)	0.11185 (16)	0.0188 (7)	
C1	0.1270 (3)	0.6469 (3)	0.17711 (19)	0.0157 (7)	
C2	0.0092 (3)	0.7377 (3)	0.1121 (2)	0.0247 (9)	
H2	−0.037697	0.757036	0.072831	0.030*	
C3	0.1287 (3)	0.7643 (3)	0.2866 (2)	0.0208 (8)	
H3A	0.147907	0.841670	0.287777	0.025*	
H3B	0.185333	0.720725	0.306306	0.025*	
C4	0.0504 (3)	0.7484 (4)	0.3328 (2)	0.0294 (9)	
H4A	−0.004342	0.794651	0.314950	0.044*	
H4B	0.074689	0.767984	0.382430	0.044*	
H4C	0.030218	0.672133	0.330718	0.044*	
C5	0.0709 (3)	0.5775 (3)	0.0507 (2)	0.0226 (8)	
H5A	0.125030	0.526128	0.061069	0.027*	
H5B	0.080639	0.620510	0.008007	0.027*	
C6	−0.0221 (3)	0.5135 (3)	0.0342 (2)	0.0235 (8)	
H6A	−0.076909	0.563972	0.032751	0.028*	
H6B	−0.025962	0.460361	0.073018	0.028*	
C7	−0.0291 (4)	0.4536 (3)	−0.0373 (3)	0.0259 (11)	
H7A	−0.021690	0.506301	−0.075669	0.031*	
H7B	0.023736	0.400339	−0.034862	0.031*	
C8	−0.1244 (3)	0.3945 (4)	−0.0558 (2)	0.0342 (10)	
H8A	−0.125073	0.354691	−0.100850	0.051*	
H8B	−0.176654	0.447436	−0.061068	0.051*	
H8C	−0.132580	0.343321	−0.017389	0.051*	
C9	0.3233 (3)	0.8062 (3)	0.1857 (2)	0.0165 (7)	
C10	0.3651 (3)	0.8906 (3)	0.2285 (2)	0.0248 (9)	
H10	0.409047	0.874911	0.270329	0.030*	
C11	0.3423 (3)	0.9983 (3)	0.2100 (3)	0.0326 (10)	
H11	0.371298	1.055455	0.239325	0.039*	
C12	0.2783 (3)	1.0229 (3)	0.1498 (3)	0.0305 (10)	
H12	0.263464	1.096466	0.137737	0.037*	
C13	0.2357 (3)	0.9401 (3)	0.1068 (2)	0.0265 (9)	
H13	0.191443	0.956758	0.065383	0.032*	
C14	0.2578 (3)	0.8322 (3)	0.1245 (2)	0.0240 (8)	
H14	0.228402	0.775645	0.094877	0.029*	
C15	0.4034 (3)	0.6293 (3)	0.12137 (19)	0.0150 (7)	
C16	0.4761 (3)	0.6959 (3)	0.1030 (2)	0.0200 (8)	
H16	0.495898	0.757864	0.131339	0.024*	
C17	0.5196 (3)	0.6717 (3)	0.0432 (2)	0.0251 (9)	
H17	0.569552	0.716459	0.031062	0.030*	
C18	0.4895 (3)	0.5818 (3)	0.0017 (2)	0.0250 (9)	
H18	0.519025	0.565114	−0.039086	0.030*	
C19	0.4167 (3)	0.5162 (3)	0.0191 (2)	0.0233 (8)	
H19	0.396371	0.455065	−0.009927	0.028*	
C20	0.3731 (3)	0.5395 (3)	0.0791 (2)	0.0184 (8)	
H20	0.323146	0.494522	0.090993	0.022*	
C21	0.4501 (3)	0.6576 (3)	0.27486 (19)	0.0168 (7)	
C22	0.5350 (3)	0.6010 (3)	0.2688 (2)	0.0224 (8)	
H22	0.545238	0.572458	0.223798	0.027*	
C23	0.6042 (3)	0.5869 (3)	0.3291 (2)	0.0307 (10)	
H23	0.661476	0.548754	0.324954	0.037*	
C24	0.5895 (4)	0.6283 (4)	0.3947 (2)	0.0350 (11)	
H24	0.636842	0.618770	0.435446	0.042*	
C25	0.5061 (3)	0.6836 (3)	0.4012 (2)	0.0314 (10)	
H25	0.496630	0.712453	0.446309	0.038*	
C26	0.4361 (3)	0.6972 (3)	0.3420 (2)	0.0232 (9)	
H26	0.378287	0.733619	0.347221	0.028*	
C27	0.3181 (3)	0.4482 (3)	0.3088 (2)	0.0181 (8)	
H27	0.369372	0.491922	0.337835	0.022*	
C28	0.3413 (3)	0.4137 (3)	0.2441 (2)	0.0170 (9)	
H28	0.406472	0.435996	0.235014	0.020*	
C29	0.3032 (3)	0.3131 (3)	0.2044 (2)	0.0200 (8)	
H29A	0.339055	0.249255	0.226038	0.024*	
H29B	0.314771	0.318570	0.154016	0.024*	
C30	0.1958 (3)	0.2946 (3)	0.2054 (2)	0.0196 (8)	
H30A	0.169955	0.250343	0.163170	0.024*	
H30B	0.187841	0.252387	0.248746	0.024*	
C31	0.1376 (3)	0.3981 (3)	0.2047 (2)	0.0199 (9)	
H31	0.086650	0.404313	0.162176	0.024*	
C32	0.1159 (3)	0.4519 (3)	0.2640 (2)	0.0219 (8)	
H32	0.052410	0.489120	0.256332	0.026*	
C33	0.1515 (3)	0.4248 (3)	0.3408 (2)	0.0280 (9)	
H33A	0.110331	0.367500	0.356808	0.034*	
H33B	0.145710	0.490050	0.370579	0.034*	
C34	0.2561 (3)	0.3857 (3)	0.3534 (2)	0.0241 (8)	
H34A	0.282156	0.394122	0.404758	0.029*	
H34B	0.258168	0.307521	0.341417	0.029*	
Rh1'	0.73530 (2)	0.95710 (2)	0.73108 (2)	0.01291 (13)	
P1'	0.85977 (7)	0.84605 (7)	0.70922 (5)	0.0131 (2)	
N1'	0.6095 (2)	0.7600 (2)	0.72286 (17)	0.0179 (7)	
N2'	0.5440 (3)	0.6938 (3)	0.68242 (18)	0.0270 (8)	
N3'	0.5873 (2)	0.8326 (3)	0.61993 (18)	0.0227 (7)	
C1'	0.6382 (3)	0.8450 (3)	0.6868 (2)	0.0172 (7)	
C2'	0.5320 (3)	0.7405 (4)	0.6201 (2)	0.0265 (9)	
H2'	0.490327	0.714373	0.579647	0.032*	
C3'	0.6345 (3)	0.7359 (3)	0.7994 (2)	0.0204 (8)	
H3'A	0.686481	0.784950	0.820734	0.024*	
H3'B	0.657881	0.660235	0.805607	0.024*	
C4'	0.5481 (3)	0.7508 (4)	0.8375 (2)	0.0268 (9)	
H4'A	0.498359	0.698649	0.818527	0.040*	
H4'B	0.523428	0.824811	0.829550	0.040*	
H4'C	0.566668	0.738388	0.888924	0.040*	
C5'	0.5869 (3)	0.9051 (3)	0.5583 (2)	0.0248 (9)	
H5'A	0.601412	0.862715	0.516605	0.030*	
H5'B	0.637271	0.960830	0.569555	0.030*	
C6'	0.4898 (3)	0.9608 (3)	0.5391 (2)	0.0271 (9)	
H6'A	0.438943	0.904899	0.533216	0.033*	
H6'B	0.478590	1.008868	0.579099	0.033*	
C7'	0.4834 (3)	1.0276 (4)	0.4706 (3)	0.0321 (10)	
H7'A	0.533190	1.084687	0.476889	0.039*	
H7'B	0.496125	0.979903	0.430813	0.039*	
C8'	0.3861 (4)	1.0804 (4)	0.4510 (3)	0.0422 (12)	
H8'A	0.336782	1.024020	0.443167	0.063*	
H8'B	0.385357	1.123027	0.407033	0.063*	
H8'C	0.373457	1.128192	0.489992	0.063*	
C9'	0.9043 (3)	0.8808 (3)	0.62603 (18)	0.0145 (7)	
C10'	0.9738 (3)	0.8152 (3)	0.6024 (2)	0.0185 (7)	
H10'	0.996074	0.752189	0.628768	0.022*	
C11'	1.0107 (3)	0.8418 (3)	0.5402 (2)	0.0237 (8)	
H11'	1.058557	0.797575	0.524508	0.028*	
C12'	0.9775 (3)	0.9331 (4)	0.5010 (2)	0.0251 (10)	
H12'	1.002655	0.951173	0.458566	0.030*	
C13'	0.9076 (3)	0.9981 (3)	0.5238 (2)	0.0238 (8)	
H13'	0.884945	1.060312	0.496729	0.029*	
C14'	0.8705 (3)	0.9723 (3)	0.5863 (2)	0.0194 (8)	
H14'	0.822517	1.016681	0.601756	0.023*	
C15'	0.8355 (3)	0.7005 (3)	0.6965 (2)	0.0170 (7)	
C16'	0.8639 (3)	0.6211 (3)	0.7476 (2)	0.0278 (9)	
H16'	0.900957	0.640444	0.791893	0.033*	
C17'	0.8379 (4)	0.5130 (3)	0.7339 (3)	0.0397 (12)	
H17'	0.857749	0.459075	0.768886	0.048*	
C18'	0.7835 (3)	0.4835 (3)	0.6698 (3)	0.0317 (10)	
H18'	0.765964	0.409788	0.660930	0.038*	
C19'	0.7548 (3)	0.5622 (3)	0.6184 (2)	0.0281 (9)	
H19'	0.717544	0.542360	0.574267	0.034*	
C20'	0.7805 (3)	0.6700 (3)	0.6317 (2)	0.0233 (8)	
H20'	0.760504	0.723625	0.596512	0.028*	
C21'	0.9652 (3)	0.8568 (3)	0.77776 (19)	0.0158 (7)	
C22'	0.9593 (3)	0.8222 (3)	0.8480 (2)	0.0215 (8)	
H22'	0.902247	0.789028	0.858255	0.026*	
C23'	1.0352 (3)	0.8358 (3)	0.9021 (2)	0.0249 (9)	
H23'	1.030375	0.810408	0.948986	0.030*	
C24'	1.1180 (3)	0.8860 (3)	0.8889 (2)	0.0262 (9)	
H24'	1.170188	0.894725	0.926353	0.031*	
C25'	1.1246 (3)	0.9237 (3)	0.8203 (2)	0.0234 (8)	
H25'	1.181102	0.959484	0.811056	0.028*	
C26'	1.0484 (3)	0.9093 (3)	0.76472 (19)	0.0167 (7)	
H26'	1.053350	0.935261	0.718008	0.020*	
C27'	0.8399 (3)	1.0917 (3)	0.7551 (3)	0.0222 (10)	
H27'	0.905815	1.073244	0.745691	0.027*	
C28'	0.8189 (3)	1.0523 (3)	0.8189 (2)	0.0230 (9)	
H28'	0.872128	1.011041	0.847644	0.028*	
C29'	0.7548 (4)	1.1113 (4)	0.8628 (3)	0.0417 (12)	
H29C	0.778535	1.099191	0.914213	0.050*	
H29D	0.758582	1.190154	0.853404	0.050*	
C30'	0.6531 (4)	1.0766 (5)	0.8477 (3)	0.0463 (13)	
H30C	0.612689	1.136581	0.861206	0.056*	
H30D	0.644127	1.013913	0.878899	0.056*	
C31'	0.6177 (3)	1.0451 (3)	0.7714 (3)	0.0247 (9)	
H31'	0.555792	1.004605	0.765066	0.030*	
C32'	0.6370 (3)	1.0957 (4)	0.7101 (3)	0.0260 (10)	
H32'	0.586200	1.085493	0.667823	0.031*	
C33'	0.6924 (3)	1.2019 (3)	0.7089 (3)	0.0380 (12)	
H33C	0.669981	1.239425	0.663110	0.046*	
H33D	0.676222	1.248843	0.747982	0.046*	
C34'	0.7989 (3)	1.1922 (3)	0.7167 (3)	0.0360 (11)	
H34C	0.827590	1.256566	0.742999	0.043*	
H34D	0.818229	1.193409	0.668401	0.043*	
F1	0.2509 (2)	0.6636 (2)	0.42532 (15)	0.0411 (7)	
F2	0.2156 (2)	0.8288 (2)	0.46740 (19)	0.0511 (8)	
F3	0.3461 (2)	0.7351 (2)	0.52034 (18)	0.0471 (7)	
F4	0.1968 (2)	0.6753 (2)	0.53083 (15)	0.0465 (7)	
B1	0.2520 (4)	0.7271 (4)	0.4853 (3)	0.0289 (10)	
F1'	0.7750 (3)	0.7124 (3)	1.05755 (18)	0.0515 (9)	0.814 (4)
F2'	0.7148 (3)	0.8192 (3)	0.9653 (3)	0.0526 (10)	0.814 (4)
F4'	0.7153 (2)	0.6401 (2)	0.95288 (17)	0.0470 (7)	
F3'	0.8557 (2)	0.7306 (3)	0.96511 (18)	0.0351 (7)	0.814 (4)
B1'	0.7619 (4)	0.7298 (5)	0.9835 (3)	0.0392 (10)	
F2"	0.7129 (11)	0.8046 (12)	1.0293 (8)	0.0544 (18)	0.186 (4)
F3"	0.7460 (12)	0.8254 (10)	0.9307 (8)	0.0406 (18)	0.186 (4)
F1"	0.8545 (8)	0.7527 (14)	1.0082 (9)	0.0499 (18)	0.186 (4)

### Atomic displacement parameters (Å^2^)

**Table:**

	*U*^11^	*U*^22^	*U*^33^	*U*^12^	*U*^13^	*U*^23^
Rh1	0.01014 (19)	0.00934 (13)	0.0149 (3)	−0.00091 (10)	0.0025 (2)	0.00020 (11)
P1	0.0133 (5)	0.0103 (4)	0.0145 (5)	−0.0028 (3)	0.0030 (4)	−0.0010 (3)
N1	0.0176 (17)	0.0156 (14)	0.0172 (15)	0.0053 (12)	0.0011 (12)	−0.0006 (12)
N2	0.0249 (19)	0.0272 (17)	0.0194 (16)	0.0122 (14)	0.0005 (14)	0.0024 (13)
N3	0.0188 (18)	0.0214 (16)	0.0158 (15)	0.0044 (12)	0.0013 (13)	−0.0042 (12)
C1	0.0143 (19)	0.0149 (17)	0.0177 (18)	−0.0008 (13)	0.0018 (14)	−0.0014 (13)
C2	0.022 (2)	0.032 (2)	0.0194 (19)	0.0125 (17)	−0.0009 (16)	0.0010 (16)
C3	0.022 (2)	0.022 (2)	0.0183 (19)	0.0040 (15)	0.0016 (16)	−0.0030 (15)
C4	0.026 (2)	0.042 (2)	0.021 (2)	0.0131 (18)	0.0056 (17)	0.0019 (17)
C5	0.025 (2)	0.026 (2)	0.0172 (18)	0.0032 (16)	0.0030 (16)	−0.0054 (15)
C6	0.017 (2)	0.033 (2)	0.0197 (19)	0.0037 (16)	−0.0004 (15)	−0.0024 (16)
C7	0.023 (2)	0.032 (2)	0.021 (2)	−0.0010 (16)	−0.0006 (18)	−0.0048 (16)
C8	0.032 (3)	0.044 (3)	0.026 (2)	−0.0048 (19)	−0.0018 (18)	−0.0045 (18)
C9	0.017 (2)	0.0140 (17)	0.0199 (19)	−0.0021 (13)	0.0089 (15)	−0.0002 (14)
C10	0.024 (2)	0.0168 (19)	0.033 (2)	−0.0014 (15)	0.0005 (17)	−0.0047 (16)
C11	0.033 (3)	0.015 (2)	0.048 (3)	−0.0015 (17)	0.001 (2)	−0.0095 (18)
C12	0.033 (3)	0.0165 (19)	0.043 (3)	0.0054 (17)	0.009 (2)	0.0015 (17)
C13	0.032 (3)	0.0224 (19)	0.025 (2)	0.0040 (16)	0.0036 (18)	0.0067 (15)
C14	0.032 (2)	0.0180 (19)	0.0216 (19)	−0.0031 (16)	0.0022 (17)	−0.0021 (14)
C15	0.0133 (18)	0.0160 (17)	0.0162 (17)	−0.0002 (13)	0.0038 (14)	−0.0001 (13)
C16	0.020 (2)	0.0176 (18)	0.0236 (19)	−0.0037 (14)	0.0062 (16)	−0.0009 (14)
C17	0.024 (2)	0.026 (2)	0.029 (2)	−0.0022 (16)	0.0146 (18)	0.0034 (16)
C18	0.032 (2)	0.026 (2)	0.020 (2)	0.0064 (18)	0.0126 (17)	0.0025 (16)
C19	0.031 (2)	0.0216 (19)	0.0178 (18)	−0.0020 (16)	0.0047 (16)	−0.0034 (14)
C20	0.020 (2)	0.0171 (18)	0.0180 (18)	0.0008 (13)	0.0024 (15)	0.0027 (13)
C21	0.019 (2)	0.0148 (17)	0.0159 (17)	−0.0074 (13)	−0.0001 (14)	0.0019 (13)
C22	0.019 (2)	0.0189 (18)	0.028 (2)	−0.0048 (14)	−0.0002 (16)	0.0038 (15)
C23	0.025 (2)	0.026 (2)	0.038 (2)	−0.0063 (17)	−0.0068 (19)	0.0117 (18)
C24	0.035 (3)	0.040 (3)	0.026 (2)	−0.019 (2)	−0.0121 (19)	0.0129 (18)
C25	0.042 (3)	0.034 (2)	0.0164 (19)	−0.020 (2)	−0.0014 (18)	0.0048 (16)
C26	0.028 (2)	0.021 (2)	0.0218 (19)	−0.0137 (16)	0.0058 (17)	−0.0002 (16)
C27	0.018 (2)	0.0145 (17)	0.0198 (18)	−0.0007 (13)	−0.0045 (15)	0.0051 (13)
C28	0.012 (2)	0.0104 (19)	0.028 (2)	0.0036 (13)	−0.0010 (16)	0.0024 (15)
C29	0.0169 (19)	0.0092 (17)	0.033 (2)	0.0027 (13)	0.0000 (16)	−0.0005 (14)
C30	0.018 (2)	0.0128 (17)	0.027 (2)	−0.0018 (14)	0.0007 (16)	−0.0032 (14)
C31	0.015 (2)	0.017 (2)	0.028 (2)	−0.0063 (15)	0.0027 (17)	0.0018 (16)
C32	0.014 (2)	0.0171 (19)	0.037 (2)	−0.0032 (13)	0.0119 (17)	0.0056 (15)
C33	0.033 (2)	0.022 (2)	0.033 (2)	−0.0047 (17)	0.0169 (19)	0.0074 (17)
C34	0.029 (2)	0.0230 (19)	0.0197 (19)	−0.0027 (16)	0.0023 (16)	0.0073 (15)
Rh1'	0.00952 (19)	0.01020 (14)	0.0189 (3)	0.00142 (10)	0.0018 (2)	0.00004 (11)
P1'	0.0108 (5)	0.0117 (4)	0.0172 (5)	0.0015 (3)	0.0035 (4)	0.0027 (3)
N1'	0.0146 (17)	0.0195 (15)	0.0196 (16)	−0.0073 (12)	0.0024 (13)	−0.0009 (12)
N2'	0.024 (2)	0.0332 (19)	0.0232 (18)	−0.0150 (15)	0.0015 (15)	−0.0073 (14)
N3'	0.0192 (18)	0.0272 (17)	0.0218 (17)	−0.0067 (13)	0.0033 (14)	−0.0012 (13)
C1'	0.0126 (18)	0.0168 (17)	0.0230 (19)	0.0017 (13)	0.0051 (15)	−0.0003 (14)
C2'	0.021 (2)	0.035 (2)	0.023 (2)	−0.0131 (17)	0.0035 (17)	−0.0049 (17)
C3'	0.019 (2)	0.0216 (19)	0.0206 (19)	−0.0056 (15)	0.0015 (15)	0.0034 (15)
C4'	0.022 (2)	0.037 (2)	0.022 (2)	−0.0073 (17)	0.0043 (16)	0.0013 (17)
C5'	0.021 (2)	0.034 (2)	0.0191 (19)	−0.0029 (16)	0.0030 (16)	0.0038 (16)
C6'	0.017 (2)	0.043 (3)	0.021 (2)	−0.0035 (17)	0.0024 (16)	0.0009 (17)
C7'	0.026 (3)	0.042 (3)	0.029 (2)	0.0028 (19)	0.007 (2)	0.0041 (19)
C8'	0.029 (3)	0.059 (3)	0.038 (3)	0.011 (2)	0.003 (2)	0.011 (2)
C9'	0.0118 (17)	0.0172 (17)	0.0147 (16)	−0.0007 (13)	0.0028 (13)	0.0028 (13)
C10'	0.018 (2)	0.0183 (18)	0.0196 (18)	−0.0001 (14)	0.0037 (15)	0.0005 (14)
C11'	0.023 (2)	0.028 (2)	0.0218 (19)	−0.0029 (16)	0.0073 (16)	−0.0039 (15)
C12'	0.030 (3)	0.030 (2)	0.017 (2)	−0.0084 (19)	0.0084 (18)	−0.0003 (17)
C13'	0.031 (2)	0.0187 (19)	0.0201 (19)	−0.0031 (16)	−0.0006 (17)	0.0067 (15)
C14'	0.019 (2)	0.0171 (17)	0.0218 (19)	−0.0005 (14)	0.0006 (15)	0.0007 (14)
C15'	0.0150 (19)	0.0118 (17)	0.026 (2)	0.0001 (13)	0.0073 (15)	0.0023 (14)
C16'	0.029 (2)	0.018 (2)	0.034 (2)	−0.0020 (16)	−0.0038 (18)	0.0045 (16)
C17'	0.043 (3)	0.016 (2)	0.056 (3)	−0.0013 (18)	−0.005 (2)	0.0131 (19)
C18'	0.034 (3)	0.0140 (18)	0.047 (3)	−0.0056 (17)	0.006 (2)	−0.0012 (17)
C19'	0.031 (2)	0.021 (2)	0.032 (2)	−0.0030 (17)	0.0067 (19)	−0.0043 (16)
C20'	0.030 (2)	0.0166 (18)	0.024 (2)	−0.0006 (15)	0.0072 (17)	0.0003 (14)
C21'	0.0162 (19)	0.0138 (17)	0.0178 (17)	0.0059 (13)	0.0042 (14)	0.0003 (13)
C22'	0.024 (2)	0.0189 (19)	0.023 (2)	0.0068 (15)	0.0079 (16)	0.0036 (15)
C23'	0.032 (2)	0.025 (2)	0.0167 (18)	0.0119 (17)	0.0006 (17)	0.0004 (15)
C24'	0.025 (2)	0.028 (2)	0.023 (2)	0.0079 (16)	−0.0055 (17)	−0.0095 (16)
C25'	0.017 (2)	0.024 (2)	0.028 (2)	0.0013 (15)	0.0008 (16)	−0.0085 (16)
C26'	0.0164 (19)	0.0173 (17)	0.0166 (17)	0.0047 (13)	0.0028 (14)	−0.0018 (13)
C27'	0.015 (2)	0.0094 (19)	0.038 (3)	−0.0018 (14)	−0.0075 (18)	0.0001 (16)
C28'	0.020 (2)	0.0191 (19)	0.027 (2)	0.0057 (14)	−0.0057 (17)	−0.0059 (15)
C29'	0.041 (3)	0.047 (3)	0.036 (3)	0.010 (2)	0.003 (2)	−0.019 (2)
C30'	0.038 (3)	0.058 (3)	0.048 (3)	0.003 (2)	0.023 (3)	−0.016 (3)
C31'	0.014 (2)	0.019 (2)	0.044 (3)	0.0024 (14)	0.0135 (18)	−0.0024 (16)
C32'	0.012 (2)	0.022 (2)	0.043 (3)	0.0080 (16)	0.0020 (19)	0.0043 (19)
C33'	0.020 (2)	0.021 (2)	0.073 (4)	0.0059 (16)	0.009 (2)	0.019 (2)
C34'	0.024 (2)	0.019 (2)	0.060 (3)	−0.0059 (16)	−0.009 (2)	0.0103 (19)
F1	0.0358 (16)	0.0561 (17)	0.0292 (14)	0.0060 (12)	−0.0029 (12)	−0.0049 (12)
F2	0.0383 (17)	0.0366 (16)	0.074 (2)	0.0045 (13)	−0.0068 (15)	−0.0002 (14)
F3	0.0286 (15)	0.0442 (16)	0.0623 (19)	0.0027 (12)	−0.0133 (14)	−0.0119 (14)
F4	0.0390 (17)	0.0674 (19)	0.0347 (15)	0.0025 (14)	0.0103 (13)	0.0050 (13)
B1	0.019 (3)	0.033 (3)	0.033 (3)	0.0067 (19)	−0.004 (2)	−0.005 (2)
F1'	0.0413 (19)	0.091 (2)	0.0230 (15)	−0.0086 (17)	0.0089 (14)	−0.0018 (15)
F2'	0.041 (2)	0.0395 (17)	0.069 (2)	0.0048 (15)	−0.0205 (18)	−0.0083 (18)
F4'	0.0335 (16)	0.0489 (17)	0.0541 (18)	−0.0048 (13)	−0.0079 (14)	0.0041 (14)
F3'	0.0273 (16)	0.0563 (18)	0.0233 (16)	−0.0085 (13)	0.0094 (12)	0.0082 (13)
B1'	0.030 (2)	0.058 (2)	0.0284 (19)	−0.0047 (18)	0.0011 (17)	0.0003 (18)
F2"	0.044 (3)	0.069 (3)	0.049 (3)	−0.007 (3)	0.000 (3)	−0.006 (3)
F3"	0.033 (3)	0.049 (3)	0.037 (3)	−0.003 (3)	−0.002 (3)	−0.004 (3)
F1"	0.039 (3)	0.073 (3)	0.037 (3)	−0.008 (3)	0.002 (3)	0.005 (3)

### Geometric parameters (Å, º)

**Table:**

Rh1—P1	2.3162 (9)	P1'—C9'	1.826 (3)
Rh1—C1	2.034 (4)	P1'—C15'	1.834 (4)
Rh1—C27	2.236 (4)	P1'—C21'	1.829 (4)
Rh1—C28	2.227 (4)	N1'—N2'	1.377 (4)
Rh1—C31	2.205 (4)	N1'—C1'	1.344 (5)
Rh1—C32	2.211 (4)	N1'—C3'	1.466 (5)
P1—C9	1.837 (4)	N2'—C2'	1.297 (5)
P1—C15	1.828 (3)	N3'—C1'	1.366 (5)
P1—C21	1.825 (4)	N3'—C2'	1.378 (5)
N1—N2	1.388 (4)	N3'—C5'	1.466 (5)
N1—C1	1.341 (4)	C2'—H2'	0.9500
N1—C3	1.467 (5)	C3'—H3'A	0.9900
N2—C2	1.297 (5)	C3'—H3'B	0.9900
N3—C1	1.368 (5)	C3'—C4'	1.517 (5)
N3—C2	1.378 (5)	C4'—H4'A	0.9800
N3—C5	1.465 (4)	C4'—H4'B	0.9800
C2—H2	0.9500	C4'—H4'C	0.9800
C3—H3A	0.9900	C5'—H5'A	0.9900
C3—H3B	0.9900	C5'—H5'B	0.9900
C3—C4	1.520 (6)	C5'—C6'	1.529 (6)
C4—H4A	0.9800	C6'—H6'A	0.9900
C4—H4B	0.9800	C6'—H6'B	0.9900
C4—H4C	0.9800	C6'—C7'	1.525 (6)
C5—H5A	0.9900	C7'—H7'A	0.9900
C5—H5B	0.9900	C7'—H7'B	0.9900
C5—C6	1.526 (6)	C7'—C8'	1.517 (6)
C6—H6A	0.9900	C8'—H8'A	0.9800
C6—H6B	0.9900	C8'—H8'B	0.9800
C6—C7	1.528 (6)	C8'—H8'C	0.9800
C7—H7A	0.9900	C9'—C10'	1.396 (5)
C7—H7B	0.9900	C9'—C14'	1.399 (5)
C7—C8	1.527 (6)	C10'—H10'	0.9500
C8—H8A	0.9800	C10'—C11'	1.391 (5)
C8—H8B	0.9800	C11'—H11'	0.9500
C8—H8C	0.9800	C11'—C12'	1.389 (6)
C9—C10	1.393 (5)	C12'—H12'	0.9500
C9—C14	1.407 (6)	C12'—C13'	1.387 (6)
C10—H10	0.9500	C13'—H13'	0.9500
C10—C11	1.397 (6)	C13'—C14'	1.397 (5)
C11—H11	0.9500	C14'—H14'	0.9500
C11—C12	1.379 (7)	C15'—C16'	1.391 (5)
C12—H12	0.9500	C15'—C20'	1.400 (6)
C12—C13	1.384 (6)	C16'—H16'	0.9500
C13—H13	0.9500	C16'—C17'	1.395 (6)
C13—C14	1.394 (5)	C17'—H17'	0.9500
C14—H14	0.9500	C17'—C18'	1.383 (7)
C15—C16	1.398 (5)	C18'—H18'	0.9500
C15—C20	1.394 (5)	C18'—C19'	1.388 (6)
C16—H16	0.9500	C19'—H19'	0.9500
C16—C17	1.394 (5)	C19'—C20'	1.390 (5)
C17—H17	0.9500	C20'—H20'	0.9500
C17—C18	1.387 (6)	C21'—C22'	1.405 (5)
C18—H18	0.9500	C21'—C26'	1.396 (5)
C18—C19	1.386 (6)	C22'—H22'	0.9500
C19—H19	0.9500	C22'—C23'	1.380 (6)
C19—C20	1.397 (5)	C23'—H23'	0.9500
C20—H20	0.9500	C23'—C24'	1.379 (6)
C21—C22	1.406 (5)	C24'—H24'	0.9500
C21—C26	1.400 (5)	C24'—C25'	1.392 (6)
C22—H22	0.9500	C25'—H25'	0.9500
C22—C23	1.399 (6)	C25'—C26'	1.400 (5)
C23—H23	0.9500	C26'—H26'	0.9500
C23—C24	1.383 (7)	C27'—H27'	1.0000
C24—H24	0.9500	C27'—C28'	1.370 (6)
C24—C25	1.383 (7)	C27'—C34'	1.506 (6)
C25—H25	0.9500	C28'—H28'	1.0000
C25—C26	1.389 (6)	C28'—C29'	1.502 (6)
C26—H26	0.9500	C29'—H29C	0.9900
C27—H27	1.0000	C29'—H29D	0.9900
C27—C28	1.379 (6)	C29'—C30'	1.488 (7)
C27—C34	1.511 (5)	C30'—H30C	0.9900
C28—H28	1.0000	C30'—H30D	0.9900
C28—C29	1.506 (5)	C30'—C31'	1.505 (7)
C29—H29A	0.9900	C31'—H31'	1.0000
C29—H29B	0.9900	C31'—C32'	1.375 (6)
C29—C30	1.539 (6)	C32'—H32'	1.0000
C30—H30A	0.9900	C32'—C33'	1.527 (6)
C30—H30B	0.9900	C33'—H33C	0.9900
C30—C31	1.517 (6)	C33'—H33D	0.9900
C31—H31	1.0000	C33'—C34'	1.497 (7)
C31—C32	1.372 (6)	C34'—H34C	0.9900
C32—H32	1.0000	C34'—H34D	0.9900
C32—C33	1.502 (6)	F1—B1	1.374 (6)
C33—H33A	0.9900	F2—B1	1.378 (6)
C33—H33B	0.9900	F3—B1	1.400 (5)
C33—C34	1.541 (6)	F4—B1	1.397 (6)
C34—H34A	0.9900	F1'—B1'	1.398 (6)
C34—H34B	0.9900	F2'—B1'	1.306 (7)
Rh1'—P1'	2.3135 (9)	F4'—B1'	1.370 (6)
Rh1'—C1'	2.038 (4)	F3'—B1'	1.421 (6)
Rh1'—C27'	2.224 (4)	B1'—F2"	1.500 (10)
Rh1'—C28'	2.223 (4)	B1'—F3"	1.538 (10)
Rh1'—C31'	2.211 (4)	B1'—F1"	1.354 (10)
Rh1'—C32'	2.201 (4)		
			
C1—Rh1—P1	91.84 (10)	C31'—Rh1'—C27'	94.36 (16)
C1—Rh1—C27	156.80 (14)	C31'—Rh1'—C28'	80.19 (16)
C1—Rh1—C28	166.63 (15)	C32'—Rh1'—P1'	154.65 (13)
C1—Rh1—C31	94.22 (15)	C32'—Rh1'—C27'	80.94 (15)
C1—Rh1—C32	86.99 (15)	C32'—Rh1'—C28'	88.73 (17)
C27—Rh1—P1	96.24 (10)	C32'—Rh1'—C31'	36.32 (17)
C28—Rh1—P1	88.28 (11)	C9'—P1'—Rh1'	112.49 (12)
C28—Rh1—C27	35.99 (15)	C9'—P1'—C15'	101.39 (17)
C31—Rh1—P1	157.34 (12)	C9'—P1'—C21'	104.06 (17)
C31—Rh1—C27	86.63 (16)	C15'—P1'—Rh1'	117.95 (12)
C31—Rh1—C28	80.88 (15)	C21'—P1'—Rh1'	113.17 (11)
C31—Rh1—C32	36.20 (15)	C21'—P1'—C15'	106.29 (17)
C32—Rh1—P1	166.31 (11)	N2'—N1'—C3'	118.2 (3)
C32—Rh1—C27	80.05 (15)	C1'—N1'—N2'	114.1 (3)
C32—Rh1—C28	96.00 (15)	C1'—N1'—C3'	127.6 (3)
C9—P1—Rh1	120.02 (12)	C2'—N2'—N1'	103.2 (3)
C15—P1—Rh1	113.30 (12)	C1'—N3'—C2'	108.2 (3)
C15—P1—C9	100.23 (16)	C1'—N3'—C5'	127.2 (3)
C21—P1—Rh1	111.25 (11)	C2'—N3'—C5'	124.6 (3)
C21—P1—C9	105.63 (17)	N1'—C1'—Rh1'	123.7 (3)
C21—P1—C15	104.88 (17)	N1'—C1'—N3'	102.9 (3)
N2—N1—C3	118.6 (3)	N3'—C1'—Rh1'	133.4 (3)
C1—N1—N2	113.9 (3)	N2'—C2'—N3'	111.6 (4)
C1—N1—C3	127.5 (3)	N2'—C2'—H2'	124.2
C2—N2—N1	102.9 (3)	N3'—C2'—H2'	124.2
C1—N3—C2	107.9 (3)	N1'—C3'—H3'A	109.6
C1—N3—C5	128.3 (3)	N1'—C3'—H3'B	109.6
C2—N3—C5	123.9 (3)	N1'—C3'—C4'	110.3 (3)
N1—C1—Rh1	123.6 (3)	H3'A—C3'—H3'B	108.1
N1—C1—N3	103.3 (3)	C4'—C3'—H3'A	109.6
N3—C1—Rh1	133.0 (3)	C4'—C3'—H3'B	109.6
N2—C2—N3	112.0 (3)	C3'—C4'—H4'A	109.5
N2—C2—H2	124.0	C3'—C4'—H4'B	109.5
N3—C2—H2	124.0	C3'—C4'—H4'C	109.5
N1—C3—H3A	109.4	H4'A—C4'—H4'B	109.5
N1—C3—H3B	109.4	H4'A—C4'—H4'C	109.5
N1—C3—C4	111.2 (3)	H4'B—C4'—H4'C	109.5
H3A—C3—H3B	108.0	N3'—C5'—H5'A	109.4
C4—C3—H3A	109.4	N3'—C5'—H5'B	109.4
C4—C3—H3B	109.4	N3'—C5'—C6'	111.1 (3)
C3—C4—H4A	109.5	H5'A—C5'—H5'B	108.0
C3—C4—H4B	109.5	C6'—C5'—H5'A	109.4
C3—C4—H4C	109.5	C6'—C5'—H5'B	109.4
H4A—C4—H4B	109.5	C5'—C6'—H6'A	109.1
H4A—C4—H4C	109.5	C5'—C6'—H6'B	109.1
H4B—C4—H4C	109.5	H6'A—C6'—H6'B	107.8
N3—C5—H5A	109.2	C7'—C6'—C5'	112.5 (3)
N3—C5—H5B	109.2	C7'—C6'—H6'A	109.1
N3—C5—C6	111.9 (3)	C7'—C6'—H6'B	109.1
H5A—C5—H5B	107.9	C6'—C7'—H7'A	109.2
C6—C5—H5A	109.2	C6'—C7'—H7'B	109.2
C6—C5—H5B	109.2	H7'A—C7'—H7'B	107.9
C5—C6—H6A	109.3	C8'—C7'—C6'	112.2 (4)
C5—C6—H6B	109.3	C8'—C7'—H7'A	109.2
C5—C6—C7	111.8 (3)	C8'—C7'—H7'B	109.2
H6A—C6—H6B	107.9	C7'—C8'—H8'A	109.5
C7—C6—H6A	109.3	C7'—C8'—H8'B	109.5
C7—C6—H6B	109.3	C7'—C8'—H8'C	109.5
C6—C7—H7A	109.2	H8'A—C8'—H8'B	109.5
C6—C7—H7B	109.2	H8'A—C8'—H8'C	109.5
H7A—C7—H7B	107.9	H8'B—C8'—H8'C	109.5
C8—C7—C6	111.9 (4)	C10'—C9'—P1'	119.2 (3)
C8—C7—H7A	109.2	C10'—C9'—C14'	119.8 (3)
C8—C7—H7B	109.2	C14'—C9'—P1'	121.0 (3)
C7—C8—H8A	109.5	C9'—C10'—H10'	119.9
C7—C8—H8B	109.5	C11'—C10'—C9'	120.1 (3)
C7—C8—H8C	109.5	C11'—C10'—H10'	119.9
H8A—C8—H8B	109.5	C10'—C11'—H11'	120.0
H8A—C8—H8C	109.5	C12'—C11'—C10'	120.1 (4)
H8B—C8—H8C	109.5	C12'—C11'—H11'	120.0
C10—C9—P1	124.6 (3)	C11'—C12'—H12'	119.9
C10—C9—C14	118.6 (3)	C13'—C12'—C11'	120.2 (4)
C14—C9—P1	116.8 (3)	C13'—C12'—H12'	119.9
C9—C10—H10	120.0	C12'—C13'—H13'	119.9
C9—C10—C11	120.0 (4)	C12'—C13'—C14'	120.2 (4)
C11—C10—H10	120.0	C14'—C13'—H13'	119.9
C10—C11—H11	119.5	C9'—C14'—H14'	120.2
C12—C11—C10	120.9 (4)	C13'—C14'—C9'	119.7 (4)
C12—C11—H11	119.5	C13'—C14'—H14'	120.2
C11—C12—H12	120.1	C16'—C15'—P1'	124.6 (3)
C11—C12—C13	119.8 (4)	C16'—C15'—C20'	118.9 (3)
C13—C12—H12	120.1	C20'—C15'—P1'	116.5 (3)
C12—C13—H13	120.0	C15'—C16'—H16'	120.0
C12—C13—C14	120.0 (4)	C15'—C16'—C17'	120.1 (4)
C14—C13—H13	120.0	C17'—C16'—H16'	120.0
C9—C14—H14	119.7	C16'—C17'—H17'	119.7
C13—C14—C9	120.7 (4)	C18'—C17'—C16'	120.7 (4)
C13—C14—H14	119.7	C18'—C17'—H17'	119.7
C16—C15—P1	118.9 (3)	C17'—C18'—H18'	120.1
C20—C15—P1	121.2 (3)	C17'—C18'—C19'	119.7 (4)
C20—C15—C16	119.9 (3)	C19'—C18'—H18'	120.1
C15—C16—H16	119.9	C18'—C19'—H19'	120.1
C17—C16—C15	120.2 (3)	C18'—C19'—C20'	119.9 (4)
C17—C16—H16	119.9	C20'—C19'—H19'	120.1
C16—C17—H17	120.2	C15'—C20'—H20'	119.6
C18—C17—C16	119.5 (4)	C19'—C20'—C15'	120.8 (4)
C18—C17—H17	120.2	C19'—C20'—H20'	119.6
C17—C18—H18	119.7	C22'—C21'—P1'	119.3 (3)
C19—C18—C17	120.6 (4)	C26'—C21'—P1'	122.0 (3)
C19—C18—H18	119.7	C26'—C21'—C22'	118.3 (4)
C18—C19—H19	119.9	C21'—C22'—H22'	119.5
C18—C19—C20	120.3 (4)	C23'—C22'—C21'	121.0 (4)
C20—C19—H19	119.9	C23'—C22'—H22'	119.5
C15—C20—C19	119.5 (4)	C22'—C23'—H23'	119.7
C15—C20—H20	120.3	C24'—C23'—C22'	120.6 (4)
C19—C20—H20	120.3	C24'—C23'—H23'	119.7
C22—C21—P1	121.7 (3)	C23'—C24'—H24'	120.2
C26—C21—P1	118.7 (3)	C23'—C24'—C25'	119.5 (4)
C26—C21—C22	118.9 (4)	C25'—C24'—H24'	120.2
C21—C22—H22	120.0	C24'—C25'—H25'	119.9
C23—C22—C21	119.9 (4)	C24'—C25'—C26'	120.3 (4)
C23—C22—H22	120.0	C26'—C25'—H25'	119.9
C22—C23—H23	119.9	C21'—C26'—C25'	120.2 (3)
C24—C23—C22	120.2 (4)	C21'—C26'—H26'	119.9
C24—C23—H23	119.9	C25'—C26'—H26'	119.9
C23—C24—H24	119.9	Rh1'—C27'—H27'	113.8
C23—C24—C25	120.2 (4)	C28'—C27'—Rh1'	72.0 (2)
C25—C24—H24	119.9	C28'—C27'—H27'	113.8
C24—C25—H25	119.8	C28'—C27'—C34'	126.5 (4)
C24—C25—C26	120.3 (4)	C34'—C27'—Rh1'	108.5 (3)
C26—C25—H25	119.8	C34'—C27'—H27'	113.8
C21—C26—H26	119.8	Rh1'—C28'—H28'	114.4
C25—C26—C21	120.5 (4)	C27'—C28'—Rh1'	72.1 (2)
C25—C26—H26	119.8	C27'—C28'—H28'	114.4
Rh1—C27—H27	113.8	C27'—C28'—C29'	122.7 (4)
C28—C27—Rh1	71.6 (2)	C29'—C28'—Rh1'	111.7 (3)
C28—C27—H27	113.8	C29'—C28'—H28'	114.4
C28—C27—C34	124.8 (3)	C28'—C29'—H29C	108.9
C34—C27—Rh1	111.8 (3)	C28'—C29'—H29D	108.9
C34—C27—H27	113.8	H29C—C29'—H29D	107.7
Rh1—C28—H28	114.4	C30'—C29'—C28'	113.5 (4)
C27—C28—Rh1	72.4 (2)	C30'—C29'—H29C	108.9
C27—C28—H28	114.4	C30'—C29'—H29D	108.9
C27—C28—C29	125.4 (4)	C29'—C30'—H30C	108.4
C29—C28—Rh1	107.5 (3)	C29'—C30'—H30D	108.4
C29—C28—H28	114.4	C29'—C30'—C31'	115.7 (4)
C28—C29—H29A	108.9	H30C—C30'—H30D	107.4
C28—C29—H29B	108.9	C31'—C30'—H30C	108.4
C28—C29—C30	113.4 (3)	C31'—C30'—H30D	108.4
H29A—C29—H29B	107.7	Rh1'—C31'—H31'	113.8
C30—C29—H29A	108.9	C30'—C31'—Rh1'	107.1 (3)
C30—C29—H29B	108.9	C30'—C31'—H31'	113.8
C29—C30—H30A	108.7	C32'—C31'—Rh1'	71.4 (2)
C29—C30—H30B	108.7	C32'—C31'—C30'	127.5 (4)
H30A—C30—H30B	107.6	C32'—C31'—H31'	113.8
C31—C30—C29	114.3 (3)	Rh1'—C32'—H32'	114.0
C31—C30—H30A	108.7	C31'—C32'—Rh1'	72.2 (2)
C31—C30—H30B	108.7	C31'—C32'—H32'	114.0
Rh1—C31—H31	113.4	C31'—C32'—C33'	124.5 (5)
C30—C31—Rh1	111.4 (3)	C33'—C32'—Rh1'	110.8 (3)
C30—C31—H31	113.4	C33'—C32'—H32'	114.0
C32—C31—Rh1	72.1 (2)	C32'—C33'—H33C	108.2
C32—C31—C30	125.8 (4)	C32'—C33'—H33D	108.2
C32—C31—H31	113.4	H33C—C33'—H33D	107.4
Rh1—C32—H32	114.0	C34'—C33'—C32'	116.2 (4)
C31—C32—Rh1	71.7 (2)	C34'—C33'—H33C	108.2
C31—C32—H32	114.0	C34'—C33'—H33D	108.2
C31—C32—C33	126.6 (4)	C27'—C34'—H34C	108.5
C33—C32—Rh1	107.6 (3)	C27'—C34'—H34D	108.5
C33—C32—H32	114.0	C33'—C34'—C27'	115.1 (4)
C32—C33—H33A	108.9	C33'—C34'—H34C	108.5
C32—C33—H33B	108.9	C33'—C34'—H34D	108.5
C32—C33—C34	113.3 (3)	H34C—C34'—H34D	107.5
H33A—C33—H33B	107.7	F1—B1—F2	110.9 (4)
C34—C33—H33A	108.9	F1—B1—F3	108.9 (4)
C34—C33—H33B	108.9	F1—B1—F4	107.9 (4)
C27—C34—C33	112.1 (3)	F2—B1—F3	110.3 (4)
C27—C34—H34A	109.2	F2—B1—F4	110.0 (4)
C27—C34—H34B	109.2	F4—B1—F3	108.7 (4)
C33—C34—H34A	109.2	F1'—B1'—F3'	104.7 (4)
C33—C34—H34B	109.2	F2'—B1'—F1'	112.3 (5)
H34A—C34—H34B	107.9	F2'—B1'—F4'	111.9 (5)
C1'—Rh1'—P1'	90.63 (10)	F2'—B1'—F3'	112.8 (5)
C1'—Rh1'—C27'	167.58 (16)	F4'—B1'—F1'	106.5 (4)
C1'—Rh1'—C28'	156.28 (15)	F4'—B1'—F3'	108.1 (4)
C1'—Rh1'—C31'	88.72 (15)	F4'—B1'—F2"	120.2 (7)
C1'—Rh1'—C32'	94.68 (17)	F4'—B1'—F3"	109.1 (7)
C27'—Rh1'—P1'	88.62 (12)	F2"—B1'—F3"	82.4 (8)
C28'—Rh1'—P1'	96.28 (10)	F1"—B1'—F4'	134.0 (9)
C28'—Rh1'—C27'	35.91 (17)	F1"—B1'—F2"	100.3 (10)
C31'—Rh1'—P1'	168.94 (12)	F1"—B1'—F3"	96.0 (9)
			
Rh1—P1—C9—C10	119.7 (3)	Rh1'—P1'—C9'—C10'	−174.5 (3)
Rh1—P1—C9—C14	−62.5 (3)	Rh1'—P1'—C9'—C14'	6.1 (3)
Rh1—P1—C15—C16	179.4 (3)	Rh1'—P1'—C15'—C16'	−104.0 (3)
Rh1—P1—C15—C20	−1.2 (3)	Rh1'—P1'—C15'—C20'	72.7 (3)
Rh1—P1—C21—C22	101.9 (3)	Rh1'—P1'—C21'—C22'	65.4 (3)
Rh1—P1—C21—C26	−68.1 (3)	Rh1'—P1'—C21'—C26'	−107.4 (3)
Rh1—C27—C28—C29	−99.2 (4)	Rh1'—C27'—C28'—C29'	−104.7 (4)
Rh1—C27—C34—C33	−14.9 (4)	Rh1'—C27'—C34'—C33'	32.5 (6)
Rh1—C28—C29—C30	−39.0 (4)	Rh1'—C28'—C29'—C30'	11.4 (5)
Rh1—C31—C32—C33	−98.7 (4)	Rh1'—C31'—C32'—C33'	−103.4 (4)
Rh1—C32—C33—C34	−42.6 (4)	Rh1'—C32'—C33'—C34'	4.0 (6)
P1—C9—C10—C11	177.3 (3)	P1'—C9'—C10'—C11'	−178.2 (3)
P1—C9—C14—C13	−177.6 (3)	P1'—C9'—C14'—C13'	178.5 (3)
P1—C15—C16—C17	178.3 (3)	P1'—C15'—C16'—C17'	176.9 (3)
P1—C15—C20—C19	−178.7 (3)	P1'—C15'—C20'—C19'	−177.1 (3)
P1—C21—C22—C23	−171.0 (3)	P1'—C21'—C22'—C23'	−175.6 (3)
P1—C21—C26—C25	172.1 (3)	P1'—C21'—C26'—C25'	174.6 (3)
N1—N2—C2—N3	−0.9 (4)	N1'—N2'—C2'—N3'	0.1 (5)
N2—N1—C1—Rh1	−178.2 (2)	N2'—N1'—C1'—Rh1'	−179.5 (3)
N2—N1—C1—N3	−0.7 (4)	N2'—N1'—C1'—N3'	0.6 (4)
N2—N1—C3—C4	65.3 (4)	N2'—N1'—C3'—C4'	−64.7 (4)
N3—C5—C6—C7	169.5 (3)	N3'—C5'—C6'—C7'	−174.0 (3)
C1—N1—N2—C2	1.0 (4)	C1'—N1'—N2'—C2'	−0.5 (4)
C1—N1—C3—C4	−112.2 (4)	C1'—N1'—C3'—C4'	110.4 (4)
C1—N3—C2—N2	0.6 (5)	C1'—N3'—C2'—N2'	0.2 (5)
C1—N3—C5—C6	115.1 (4)	C1'—N3'—C5'—C6'	−112.4 (4)
C2—N3—C1—Rh1	177.2 (3)	C2'—N3'—C1'—Rh1'	179.6 (3)
C2—N3—C1—N1	0.1 (4)	C2'—N3'—C1'—N1'	−0.5 (4)
C2—N3—C5—C6	−64.8 (5)	C2'—N3'—C5'—C6'	65.1 (5)
C3—N1—N2—C2	−176.8 (3)	C3'—N1'—N2'—C2'	175.2 (3)
C3—N1—C1—Rh1	−0.6 (5)	C3'—N1'—C1'—Rh1'	5.3 (5)
C3—N1—C1—N3	176.9 (3)	C3'—N1'—C1'—N3'	−174.6 (3)
C5—N3—C1—Rh1	−2.6 (6)	C5'—N3'—C1'—Rh1'	−2.5 (6)
C5—N3—C1—N1	−179.8 (3)	C5'—N3'—C1'—N1'	177.3 (3)
C5—N3—C2—N2	−179.6 (4)	C5'—N3'—C2'—N2'	−177.7 (4)
C5—C6—C7—C8	−176.9 (4)	C5'—C6'—C7'—C8'	178.6 (4)
C9—P1—C15—C16	50.2 (3)	C9'—P1'—C15'—C16'	132.8 (4)
C9—P1—C15—C20	−130.3 (3)	C9'—P1'—C15'—C20'	−50.5 (3)
C9—P1—C21—C22	−126.3 (3)	C9'—P1'—C21'—C22'	−172.2 (3)
C9—P1—C21—C26	63.7 (3)	C9'—P1'—C21'—C26'	15.1 (3)
C9—C10—C11—C12	0.3 (6)	C9'—C10'—C11'—C12'	−0.8 (6)
C10—C9—C14—C13	0.4 (6)	C10'—C9'—C14'—C13'	−0.9 (6)
C10—C11—C12—C13	0.1 (7)	C10'—C11'—C12'—C13'	0.1 (6)
C11—C12—C13—C14	−0.2 (7)	C11'—C12'—C13'—C14'	0.2 (6)
C12—C13—C14—C9	0.0 (6)	C12'—C13'—C14'—C9'	0.2 (6)
C14—C9—C10—C11	−0.5 (6)	C14'—C9'—C10'—C11'	1.2 (6)
C15—P1—C9—C10	−115.7 (3)	C15'—P1'—C9'—C10'	−47.6 (3)
C15—P1—C9—C14	62.2 (3)	C15'—P1'—C9'—C14'	133.0 (3)
C15—P1—C21—C22	−20.9 (3)	C15'—P1'—C21'—C22'	−65.6 (3)
C15—P1—C21—C26	169.1 (3)	C15'—P1'—C21'—C26'	121.6 (3)
C15—C16—C17—C18	0.8 (6)	C15'—C16'—C17'—C18'	−0.3 (7)
C16—C15—C20—C19	0.8 (6)	C16'—C15'—C20'—C19'	−0.3 (6)
C16—C17—C18—C19	0.0 (6)	C16'—C17'—C18'—C19'	0.2 (7)
C17—C18—C19—C20	−0.4 (6)	C17'—C18'—C19'—C20'	−0.2 (7)
C18—C19—C20—C15	0.0 (6)	C18'—C19'—C20'—C15'	0.2 (6)
C20—C15—C16—C17	−1.2 (6)	C20'—C15'—C16'—C17'	0.3 (6)
C21—P1—C9—C10	−7.0 (4)	C21'—P1'—C9'—C10'	62.6 (3)
C21—P1—C9—C14	170.9 (3)	C21'—P1'—C9'—C14'	−116.8 (3)
C21—P1—C15—C16	−59.1 (3)	C21'—P1'—C15'—C16'	24.3 (4)
C21—P1—C15—C20	120.4 (3)	C21'—P1'—C15'—C20'	−159.0 (3)
C21—C22—C23—C24	0.0 (6)	C21'—C22'—C23'—C24'	1.5 (6)
C22—C21—C26—C25	1.9 (5)	C22'—C21'—C26'—C25'	1.8 (5)
C22—C23—C24—C25	0.2 (6)	C22'—C23'—C24'—C25'	0.4 (6)
C23—C24—C25—C26	0.6 (6)	C23'—C24'—C25'—C26'	−1.1 (6)
C24—C25—C26—C21	−1.6 (6)	C24'—C25'—C26'—C21'	0.0 (5)
C26—C21—C22—C23	−1.1 (5)	C26'—C21'—C22'—C23'	−2.6 (5)
C27—C28—C29—C30	41.5 (5)	C27'—C28'—C29'—C30'	93.5 (6)
C28—C27—C34—C33	−97.1 (5)	C28'—C27'—C34'—C33'	−48.4 (7)
C28—C29—C30—C31	33.6 (5)	C28'—C29'—C30'—C31'	−34.1 (7)
C29—C30—C31—Rh1	−9.9 (4)	C29'—C30'—C31'—Rh1'	38.3 (5)
C29—C30—C31—C32	−92.8 (5)	C29'—C30'—C31'—C32'	−41.1 (7)
C30—C31—C32—Rh1	103.9 (4)	C30'—C31'—C32'—Rh1'	97.7 (5)
C30—C31—C32—C33	5.2 (7)	C30'—C31'—C32'—C33'	−5.7 (7)
C31—C32—C33—C34	37.3 (6)	C31'—C32'—C33'—C34'	86.2 (6)
C32—C33—C34—C27	39.0 (5)	C32'—C33'—C34'—C27'	−25.1 (7)
C34—C27—C28—Rh1	104.2 (4)	C34'—C27'—C28'—Rh1'	100.0 (4)
C34—C27—C28—C29	5.0 (6)	C34'—C27'—C28'—C29'	−4.7 (7)

### Hydrogen-bond geometry (Å, º)

**Table:**

*D*—H···*A*	*D*—H	H···*A*	*D*···*A*	*D*—H···*A*
C2—H2···F3′^i^	0.95	2.37	3.263 (5)	157
C2—H2···F1"^i^	0.95	1.81	2.722 (13)	160
C17—H17···F2"^ii^	0.95	2.30	3.229 (17)	165
C2′—H2′···F3	0.95	2.19	3.002 (5)	142
C13′—H13′···F1"^iii^	0.95	2.36	3.163 (17)	142

Symmetry codes: (i) *x*−1, *y*, *z*−1; (ii) *x*, *y*, *z*−1; (iii) *x*, −*y*+2, *z*−1/2.
